# Physical Exercise Improves Glycemic and Inflammatory Profile and Attenuates Progression of Periodontitis in Diabetic Rats (HFD/STZ)

**DOI:** 10.3390/nu10111702

**Published:** 2018-11-07

**Authors:** Eric Francelino Andrade, Viviam de Oliveira Silva, Natália Oliveira de Moura, Renata de Carvalho Foureaux, Débora Ribeiro Orlando, Rodrigo Ferreira de Moura, Luciano José Pereira

**Affiliations:** 1 Department of Veterinary Medicine, Federal University of Lavras, Lavras, MG 37200-000, Brazil; ericfrancelinoandrade@gmail.com (E.F.A.); vivian_osbio@yahoo.com.br (V.d.O.S.); natioliveira.ef@gmail.com (N.O.d.M.); renatafoureaux@yahoo.com.br (R.d.C.F.); deboraribeiro.orlando@gmail.com (D.R.O.); 2 Institute of Agricultural Sciences, Federal University of Jequitinhonha and Mucuri Valleys, Unaí, MG 38610-000, Brazil; 3 Department of Research and Scientific Iniciation, Centro Universitário Atenas, Paracatu, MG 38600-000, Brazil; 4 Department of Health Sciences, Federal University of Lavras, Lavras, MG 37200-000, Brazil; rodrigo.moura@dsa.ufla.br

**Keywords:** chronic periodontitis, physical training, metabolic disorder, alveolar bone loss

## Abstract

The authors aimed to evaluate the effects of physical exercise on the metabolism and progression of periodontal disease (PD), induced by ligature in diabetic rats induced by high fat diet and streptozotocin (HFD/STZ). Diabetes Mellitus (DM) was induced by four weeks of a hyperlipidic diet associated with a single low-dose of streptozotocin (35 mg/kg/animal). The exercise groups swam for 60 min/day for eight weeks (five times/week). In the last two weeks of exercise, a ligature was placed around the right and left mandibular first molars. The authors determined alveolar bone loss by morphometry. Blood biochemical profile and serum levels of IL-10 and TNF-α were evaluated by colorimetric and enzyme-linked immunosorbent assays (ELISA), respectively. The diabetic animals subjected to exercise showed decreased alveolar bone loss, lower glycemia, triacylglycerols and glycosylated hemoglobin levels than the controls. Total cholesterol and its fractions (High density lipoprotein—HDL-c, Low density lipoprotein—LDL-c and Very low density lipoprotein—VLDL-c) remained similar among the groups. Animals with PD showed higher levels of TNF-α and lower levels of IL-10, when compared to animals without PD. In diabetic animals with PD, physical exercise decreased TNF-α levels and increased IL-10 levels as well as the IL10/TNF-α ratio. In conclusion, eight weeks of physical exercise improved glycemic control and systemic inflammatory profile, and attenuated alveolar bone loss in rats with DM and PD.

## 1. Introduction

Diabetes Mellitus (DM) is a metabolic disorder characterized mainly by chronic hyperglycemia in which pancreas beta-cells produces little or no insulin (type 1 DM); or insulin resistance (DM type 2) [1]. It is estimated that over 285 million people worldwide are diabetic, especially type 2 (90%) [2]. Type 2 diabetes incidence is increasing due to the high prevalence of obesity and overweight in both developing and developed countries [3].

Uncompensated diabetic patients commonly exhibit cardiovascular, renal and neural complications and are also more prone to develop severe oral diseases, including periodontal disease (PD) [4]. PD is generally initiated by a bacterial biofilm deposition over tooth surfaces, promoting inflammatory gum response and eventually alveolar bone resorption [5]. PD is considered the sixth most prevalent comorbidity of DM [6], and a bidirectional relationship exists between both diseases. PD cytokines impair glycemic control and contribute to insulin resistance, while hyperglycemia accelerates the progression of alveolar bone loss [7,8].

Conventional treatment of diabetes (especially type 2) involves changing habits, such as diet control and physical activity. Eventually, some patients need to use oral hypoglycemic agents and/or insulin, depending on the severity of the disease [9,10]. Studies investigating the effects of regular physical activity on diabetic patients with periodontal disease are scarce, especially when inflammatory and metabolic aspects are evaluated simultaneously. Some evidence indicates that physically active individuals are less likely to develop more severe forms of PD, when compared to sedentary individuals [11]. Considering physical exercises can modulate the immune/inflammatory response [12,13] and improve glycemic control [14], exercise may become an interesting strategy in the treatment of patients affected by both diseases.

Therefore, the purpose of the present study was to evaluate the effects of physical exercise on the inflammatory/metabolic profile and on the progression of alveolar bone loss in diabetic rats (HFD/STZ) induced to periodontal disease.

## 2. Material and Methods

### 2.1. Animals

This study was approved by the Ethics Committee on Animal Use of Universidade Federal de Lavras (CEUA protocol 002/2015). The animals were housed under standard conditions and the room was maintained on a cycle of 12-h light and 12-h darkness, at a temperature of 23 °C to 25 °C. The number of animals per group was kept at minimum for ethical reasons, but still enough to reach statistical significance. The sample size was determined to provide 80% power to recognize a significant difference of 20% in alveolar bone loss among groups and a standard deviation of 15% with a 95% confidence interval (α = 0.05).

We used healthy adult male Wistar rats (*Rattus norvegicus albinos*)—from the Animal Laboratory of the Federal University of Lavras (UFLA). At the beginning of the experiment, the animals weighed 230.8 ± 22.8 g. They were randomly distributed in a 2 × 2 × 2 factorial scheme (diabetic or not, induced to periodontal disease or not, submitted to physical training or not), where each group consisted of five rodents (Table 1). Initially, the rats were subjected to seven days of acclimatization in polypropylene boxes (dimensions 41 × 34 × 17.5 cm), containing wood shavings (for absorbing urine and water). Five animals were placed in each box. A high-fat diet and water were provided ad libitum throughout the experiment.

### 2.2. Induction of Diabetes Mellitus

We induced DM according to the protocol described by Wang et al. [15]. This model reproduces advanced stages of type 2 DM in humans [16,17]. A high fat diet (HFD), consisting of 25% fat, 48% carbohydrates and 20% proteins, was given to the animals for four weeks in DM groups. After 28 days, a low dose of streptozotocin (35 mg/kg body weight) dissolved in citrate buffer (pH = 4.5) was injected intraperitoneally in each animal. Rats with blood glucose higher than 200 mg/dL after 48 h were considered diabetic [15]. Rats presenting blood samples below this level were excluded from the following phases of the experiment. Glycaemia was checked weekly to ensure that diabetes was not reversed. Rodents from the other groups (not DM) received commercial feed (12% fat, 60% carbohydrate and 28% protein) and only citrate buffer solution injection intraperitoneally. Both DM and non-diabetic animals received their respective diets until the end of the experimental period.

### 2.3. Physical Training

After induction of diabetes (28 days of high fat diet and 2 days of DM induction = 30 days), we started exercise acclimatization. Initially, the animals from groups 5 to 8 stayed two hours/day for seven days in a polyethylene tank (500 L) containing five centimeters of water (32 ± 2 °C) to reduce stress against the aquatic environment (without causing changes from physical training) [18].

In the following week, the animals started swimming sessions with progressive time increments. This phase consisted of swimming without load, in 50 cm of water (to avoid animal tail contact with the bottom of the tank), where the animals swam for 10 min in the first day, increasing 10 min daily until the end of the sixth day, when each animal was swimming 60 uninterrupted minutes without load [19,20].

In the subsequent eight weeks, trained groups swam daily for 60 min, five times a week with 5% of their body weight load [21]. This load was intended to improve the animals’ endurance capacity, characterizing moderate intensity of aerobic exercise [19]. After each training session, the animals were dried with absorbent towels before returning to their boxes, as proposed by the American Physiological Society [22].

### 2.4. Periodontal Disease Induction

**Figure 1:**
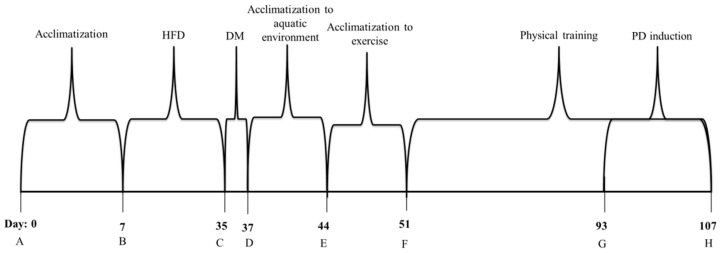
Flowchart of the experimental design. **A**—Acclimatization phase. **B**—High fat diet induction for diabetic animals and commercial feeding by nondiabetic rats. **C**—Injection of streptozotocin (35 mg/kg) for diabetic groups. **D**—Confirmation of diabetes induction (glycaemia greater than 200 mg/dL) and acclimatization to the aquatic environment for trained groups. **E**—Acclimatization to exercise. **F**—Physical training. **G**—Periodontal disease induction (ligature around lower jaw first molars). **H**—End of experiment. Euthanasia and tissue collection. HFD—High fat diet. DM—*Diabetes Mellitus*. PD—Periodontal disease.

In the last 2 weeks of training, animals from PD groups received ligatures around each lower jaw first molars (right and left) [23]. Before placing the ligature, the animals were anesthetized using an intramuscular injection of 13 mg/kg of xilazine hydrochloride 10% and 80 mg/kg of ketamine base [23]. They were placed on an operating table containing an apparatus to keep their mouths open throughout the procedure. A cotton thread was placed around the first molar of each animal’s jaw [23]. Animals from training groups rested 24 h before and 24 h after ligature placement to induce better adaptation. Those rats then returned to their normal training routine. The ligature stayed in place during 14 days (Figure 1) until euthanasia. PD induction time was based in inflammatory markers peak associated with ligature protocol [24,25].

### 2.5. Euthanasia and Collection of Biological Material

The animals fasted for eight hours before euthanasia. We used cardiac puncture under anesthesia (Thiopental Sodium 50 mg/kg i.p.). Blood samples were collected for analysis of glycosylated hemoglobin (HbA1c) (Labtest Diagnostica^®^, Belo Horizonte, Brazil) and biochemical parameters: glucose, triacylglycerol (TAG), high density lipoproteins (HDL) and total cholesterol (TC) using specific commercial kits (Gold Analisa Diagnósticos^®^, Belo Horizonte, Brazil), as described by Amr and Abeer [26]. The levels of low-density lipoproteins (LDL) were obtained through the Friedewald equation: Cholesterol LDL-C = Cholesterol Total—HDL-C-(TAG/5) [27].

### 2.6. TNF-α e IL-10 Measurement

Serum concentrations of TNF-α (Millipore, Darmstadt, Germany) and IL-10 (Sigma, St. Louis, MO, USA) were evaluated by an enzyme-linked immunosorbent assay (ELISA), using spectrophotometer optical density of 450 nm (Epoch, BioTeck Instruments Inc., Winooski, VT, USA).

### 2.7. Evaluation of Alveolar Bone Loss (ABL)

We submerged and defleshed the right side of the jaws in hydrogen peroxide for 24 h. This was followed by the cleaning, drying and staining of the specimens with 1% blue methylene [28]. Alveolar bone loss was evaluated in digital images obtained through stereomicroscopy (Leica M205A; Leica Microsystems, Wetzlar, Germany). We performed linear measurements of the distance between the cemento-enamel junction and the bone crest of each vestibular root face using Image J software (Bethesda, MD, USA) by a blind experienced examiner [28]. Alveolar Bone Loss (ABL) was determined by the mean of the three roots measurements.

### 2.8. Statistical Analyzes

Data were submitted for analysis of variance (ANOVA), and means were compared by F test at 5%. The analyses were carried out using the statistical program SISVAR version 5.3 [29].

## 3. Results

Blood glucose levels and HbA1c were higher in diabetic animals (*p* < 0.05). Animals with PD also showed higher values for HbA1c and blood glucose (*p* < 0.05), when compared to animals without PD. Physical training decreased blood glucose levels and HbA1c in diabetic animals with and without PD in relation to their respective non-trained groups (*p* < 0.05—Table 2).

Physical training decreased TAG serum concentrations in diabetic animals with and without PD (*p* < 0.05). The concentrations of total cholesterol, HDL-c, LDL-c and VLDL-c remained similar among groups (Table 3).

**Figure 2:**
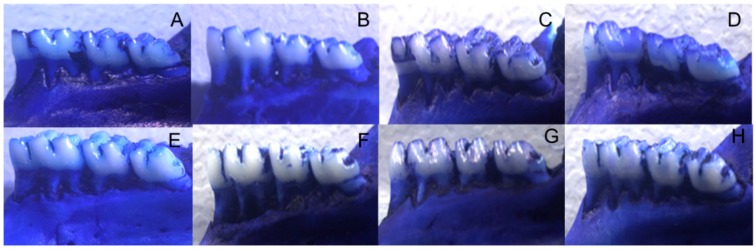
Alveolar Bone Loss (ABL) in trained or non-trained diabetic and non-diabetic rats submitted or not to ligature induction of periodontal disease and groups: (**A**)—non-trained animals without DM or PD. (**B**)—non-trained animals with PD. (**C**)—trained animals without DM or PD. (**D**)—trained animals with PD. (**E**)—type 2 diabetic (HFD/STZ) animals. (**F**)—type 2 diabetic (HFD/STZ) animals with PD. (**G**)—type 2 diabetes (HFD/STZ) trained animals. (**H**)—trained animals with type 2 diabetes (HFD/STZ) and PD.

Alveolar Bone Loss (ABL) was higher in non-trained diabetic animals with PD when compared to controls without PD or without DM (*p* < 0.05). Animals submitted to physical training presented lower ABL than those from non-trained groups (*p* < 0.05—Table 4 and Figure 2).

Animals with PD, independently of DM, showed higher values of serum TNF-α and lower values of IL-10 than those without PD (*p* < 0.05—Table 5). Physical training promoted reduction in TNF-α and increase in IL-10 levels (*p* < 0.05—Table 5). TNF-α/IL10 ratio increased in animals with PD and DM whereas IL10/TNF-α ratio decreased (*p* < 0.05). Physical training increased IL10/TNF-α ratio (*p* < 0.05—Table 5).

## 4. Discussion

Physical training improved glycemic, inflammatory and alveolar bone status in animals induced to DM and PD. PD increased both glycaemia and HbA1c levels even in animals without DM and the presence of DM altered alveolar bone integrity even in animals not submitted to ligature. The DM induction model used in the present study was designed to replicate type 2 DM in humans. The pathogenesis of type 2 DM involves genetic and environmental factors that promote insulin resistance and as a consequence reduced glucose tolerance [30]. Impaired insulin secretion may also occur due to β-cell overload [31]. Type 2 DM is associated with obesity and the secretion of proinflammatory cytokines by adipocytes (especially central obesity), which contribute to insulin resistance and β-cells injury (high levels of reactive oxygen species - ROS), compromising insulin action [10,31]. A high-fat diet initiates in animal models a similar mechanism of insulin resistance, typical of the initial stages of type 2 DM in humans. The low-dose injection of streptozotocin increase β-cell damage compromising insulin secretion [32]. Animal models of type 2 DM include naturally occurring mutations or genetic manipulation to induce congenital obesity [32]. Alternatively, obesity can be induced by high fat feeding [33]. We decided to use the HFD/STZ model to reflect the human condition where obesity is closely linked to unfavorable food ingestion/caloric waste behavior [32].

Diabetes and periodontal disease have a two-way relationship. Diabetes is associated with an increased risk of periodontitis; and glucose blood levels tend to be increased in patients with periodontal disease [34]. Both diabetes and periodontal disease involve elevated levels of inflammatory mediators such as interleukin-1β (IL-1β); tumour necrosis factor-α (TNFα), accumulation of reactive oxygen species (ROS) and advanced glycation end products (AGEs) [35]. Thus, inadequate glycemic control is associated with the increased prevalence and severity of periodontitis and severe periodontitis compromises glycemic control [34].

Improvement in glycaemia and HbA1c with exercise is a commonly reported result [36,37]. Physical exercise is one of the most recommended non-pharmacological strategies for glycemic control in individuals with DM [38]. Mechanisms involved in this improvement are related to increased glucose uptake during and after exercise, mainly through muscle tissue, as well as the decrease in insulin resistance by adipocytes [39,40]. In diabetic patients, both acute and chronic exercise favors circulating glucose consumption and uptake [41]. In addition, the increased uptake of circulating lipids, such as TAGs, also contribute to decrease insulin resistance [42]. In this study, the diabetic trained animals presented lower concentrations of TAGs when compared to the non-trained ones.

The presence of PD increased both glycaemia and HbA1c levels in rats not induced to DM, confirming the bidirectional relationship between DM and PD [43]. This behavior was observed in previous studies using both type 1 (single dose of strepozotocin) [44,45] and type 2 (obese diabetic Zucker rats) [46] using ligature-induced periodontitis models.

The induction of periodontal disease with ligature is extensively reported in the literature also due to its similarity to chronic periodontitis in humans—e.g., alveolar bone resorption depends on oral bacterial load and gingival tissue becomes infiltrated with inflammatory cells [47]. This model is considered suitable for the analysis of gingival tissue inflammation with changes related to systemic diseases [48]. Alveolar bone loss can be observed within seven days [49]. Animal models of periodontal disease are very useful to elucidate molecular pathways involved in new treatment strategies. Besides, in human studies, it is difficult to control environmental and social variables to establish a cause effect measurement of treatment outcomes [49]. Ligature placement for 14 days results in marked alveolar bone loss [23,44,50,51]. When this ligature is placed in diabetic animals, alveolar bone loss tends to be worse, as observed in our study [44]. Previous studies of our group [39,40] showed alveolar bone loss and increased levels of TNF-α in diabetic and non-diabetic rats using ligature-induced PD model. Treatment with beta-glucans decreased TNF-α in diabetic animals [44] and physical training was able to reduce gingival TNF-α/IL-10 ratio expression [40]. The increase in pro-inflammatory cytokines derived from PD (such as TNF-α) suppresses the action of insulin by preventing the binding of this hormone to its receptor [52]. TNF-α can stimulate the phosphorylation of serine residues of the insulin receptor, impairing its capacity of signaling transduction and consequent activation of the intracellular cascade that culminates with GLUT-4 expression [53]. According to this mechanism, we observed that animals with PD presented higher levels of TNF-α and poorer glycemic control than those without PD. Similarly, non-trained diabetic animals presented higher levels of circulating TNF-α, in comparison with those groups submitted to exercise. We also observed an increase in TNF-α levels in non-trained animals with PD. In addition, a rise in local pro-inflammatory cytokines, especially TNF-α, impairs bone remodeling and accelerates the destruction of periodontal tissues [54,55]. Physical training modulated this cytokine. It is reported that exercise stimulates the production of myokines with local and systemic anti-inflammatory effects [56,57]. In addition, exercise promotes the inhibition of TNF-α, which is a key cytokine in the inflammatory process and in insulin resistance [58]. The mechanisms of this inhibition probably involve the increase in IL-10 concentrations, which exerts an inhibitory effect on TNF-α elevation [58], as observed in the present study.

A reduction in serum IL-10 concentration was observed in diabetic animals compared to non-diabetic animals. This decrease in IL-10 levels is often observed in patients with PD [59,60], and it is associated with insulin resistance [53]. Interestingly, this fact was not observed in animals submitted to exercise. Exercise is able to alter the phenotype of resident macrophages in the muscle that change from pro-inflammatory to anti-inflammatory profile [58], increasing IL-10 and inhibiting TNF-α expression [58]. This fact may be related to insulin resistance mediated by this cytokine [61]. The improvement of inflammatory profile promoted by physical exercise is thus associated with better glycemic regulation that goes beyond the increase of insulin-independent glucose uptake by skeletal muscle.

Improved glycemic control in diabetic patients may attenuate the progression of bone loss observed in PD [34]. In this study, ABL was lower in trained diabetic animals, associated with the decrease in glycaemia and HbA1c levels. However, other factors may be involved in the attenuation of ABL in physically trained animals, since this improvement was also observed in non-diabetic animals with PD. Reduction of oxidative stress [62] and the improvement of inflammatory profile are also related to regular moderate physical activity [50]. This can be observed by the TNF-α/IL-10 and IL-10/TNF-α ratios in trained animals. The TNF-α/IL-10 ratio is an indicator of pro-inflammatory status, and the higher this ratio, the greater is the patient’s pro-inflammatory profile (the IL-10/TNF-α ratio indicates the inverse). This same training protocol induced increased levels of Il-10 (anti-inflammatory citokyne) and decreased levels of TNF-α in gum tissues of rats induced to ligature [50].

## 5. Conclusions

Eight weeks of physical training improved glycemic control and systemic inflammatory profile, and attenuated alveolar bone loss in rats with DM and PD.

## Figures and Tables

**Table 1:** Distribution of Wistar rats among experimental groups.

Experimental Groups	*n*
Group 1—non-trained animals without DM or PD	5
Group 2—non-trained animals with PD	5
Group 3—trained animals without DM or PD	5
Group 4—trained animals with PD	5
Group 5—type 2 diabetic (HFD/STZ) animals	5
Group 6—type 2 diabetic (HFD/STZ) animals with PD	5
Group 7—type 2 diabetes (HFD/STZ) trained animals	5
Group 8—trained animals with type 2 diabetes (HFD/STZ) and PD	5

DM—diabetes mellitus induced by high fat diet and one single intraperitoneal injection of streptozotocin (35 mg/kg). PD—Periodontal disease induced by ligature in both mandibular first molars. HFD/STZ—Diabetes induction by High Fat Diet/Streptozotocin. *n*—Number of rats per group.

**Table 2:** Blood glucose and HbA1c concentrations of type 2 diabetic rats (HFD/STZ) with ligature-induced periodontal disease after eight weeks of physical training.

Diabetes	Periodontal Disease	Physical Training	
		-	+
HbA1c (mg/dL)			
- *	-	2.66 (0.20) a	2.70 (0.16)
	+	3.18 (0.29) b	2.74 (0.23)
+	-	9.34 (0.35) x	8.86 (0.15) y
	+	9.70 (0.28) x	9.06 (0.26) y
Glucose (mg/dL)			
- *	-	98.95 (2.08) a	115.02 (5.03)
	+	121.91 (5.68) bx	102.91 (7.70) y
+	-	225.30 (11.21) x	202.24 (7.08) y
	+	240.38 (13.97) x	209.83 (15.03) y

* Difference between groups with and without diabetes by the F test (*p* < 0.05). a,b Different letters in the columns indicate differences between groups with and without periodontal disease (F test—*p* < 0.05). x,y Different letters in the lines indicate differences between the groups with and without physical training (F test—*p* < 0.05). HbA1c—Glycated hemoglobin. “-“—Without. “+”—With.

**Table 3:** Serum concentrations of triacylglycerols, total cholesterol, HDL-c, LDL-c and VLDL-c of type 2 diabetic rats (HFD/STZ) with ligature-induced periodontal disease after eight weeks of physical training.

Diabetes	Periodontal Disease	Physical Training	
		-	+
Triacylglycerols (mg/dL)			
-	-	100.35 (17.84)	94.79 (16.58)
	+	110.16 (5.51)	100.53 (9.83)
+	-	108.63 (10.97) x	90.54 (7.33) y
	+	116.78 (11.59) x	96.24 (5.10) y
Total Cholesterol (mg/dL)			
-	-	87.94 (11.27)	79.95 (12.08)
	+	95.15 (8.56)	80.50 (8.61)
+	-	85.55 (14.04)	81.42 (8.56)
	-	88.33 (6.54)	82.28 (10.82)
HDL-c (mg/dL)			
-	-	34.52 (4.00)	37.70 (2.43)
	+	39.06 (1.49)	36.29 (3.39)
+	-	36.84 (4.89)	31.73 (5.58)
	-	33.09 (2.63)	37.93 (4.52)
LDL-c (mg/dL)			
-	-	33.30 (8.36)	22.25 (5.57)
	+	36.45 (4.19)	26.50 (5.83)
+	-	28.95 (11.39)	28.73 (8.47)
	-	32.38 (10.45)	23.09 (10.23)
VDL-C (mg/dL)			
-	-	20.11 (3.60)	19.99 (4.08)
	+	21.43 (2.46)	17.70 (3.53)
+	-	19.75 (2.65)	20.95 (3.04)
	-	22.64 (2.80)	21.24 (5.00)

x,y Different letters in the lines indicate differences between the groups with and without physical training (F test—*p* < 0.05). HDL-c—High Density Lipoprotein. LDL-c—Low Density Lipoprotein. VLDL-c—Very Low Density Lipoprotein. “-“—Without. “+”—With.

**Table 4:** Alveolar bone loss of type 2 diabetic rats (HFD/STZ) with ligature-induced periodontal disease submitted to eight weeks of physical training.

Diabetes	Periodontal Disease	Physical Training	
		-	+
Alveolar bone loss (mm)			
-	-	0.90 (0.04) *a	0.94 (0.10)
	+	1.13 (0.09) bx	1.02 (0.10) y
+	-	1.03 (0.04) *ax	0.88 (0.05) ay
	-	1.29 (0.06) bx	1.06 (0.08) by

* Difference between groups with and without diabetes by the F test (*p* < 0.05). a,b Different letters in the columns indicate differences between groups with and without periodontal disease (F test—*p* < 0.05). x,y Different letters in the lines indicate differences between the groups with and without physical training (F test—*p* < 0.05). “-“—Without. “+”—With.

**Table 5:** Circulating cytokines (TNF-α and IL10), TNF-α/IL10 and IL10/TNF-α ratio in type 2 diabetic rats (HFD/STZ) with ligature-induced periodontal disease submitted to eight weeks of physical training.

Diabetes	Periodontal Disease	Physical Training	
		-	+
TNF-α (pg/mL)			
-	-	5.93 (0.86) *a	6.11 (1.41)
	+	8.66 (0.36) b	6.86 (0.24)
+	-	8.42 (1.04) a	7.20 (0.46)
	-	10.77 (3.02) bx	7.10 (0.54) y
IL-10 (pg/mL)			
- *	-	14.27 (0.46) *a	16.32 (0.42)
	+	10.18 (0.33) b	13.69 (1.48)
+	-	9.61 (1.92)	11.24 (1.21)
	-	9.62 (2.26)	12.86 (3.42)
TNF-α/IL10 ratio			
- *	-	0.41 (0.06) *a	0.37 (0.08)
	+	0.85 (0.03) b	0.51 (0.06)
+	-	0.98 (0.32)	0.64 (0.03)
	-	1.48 (1.00) x	0.60 (0.12) y
IL10/TNF-α ratio			
- *	-	2.41 (0.42) *a	2.67 (0.70) *a
	+	1.17 (0.05) b	1.99 (0.26) b
+	-	1.14 (0.30)	1.56 (0.09)
	-	0.89 (0.35) x	1.81 (0.69) y

* Difference between groups with and without diabetes by the F test (*p <* 0.05). a,b Different letters in the columns indicate differences between groups with and without periodontal disease (F test—*p <* 0.05). x,y Different letters in the lines indicate differences between the groups with and without physical training (F test—*p <* 0.05). “-“—Without. “+”—With.
